# Botulinum Neurotoxin Serotype a Specific Cell-Based Potency Assay to Replace the Mouse Bioassay

**DOI:** 10.1371/journal.pone.0049516

**Published:** 2012-11-21

**Authors:** Ester Fernández-Salas, Joanne Wang, Yanira Molina, Jeremy B. Nelson, Birgitte P. S. Jacky, K. Roger Aoki

**Affiliations:** Department of Biological Sciences, Allergan Inc., Irvine, California, United States of America; CNRS, France

## Abstract

Botulinum neurotoxin serotype A (BoNT/A), a potent therapeutic used to treat various disorders, inhibits vesicular neurotransmitter exocytosis by cleaving SNAP25. Development of cell-based potency assays (CBPAs) to assess the biological function of BoNT/A have been challenging because of its potency. CBPAs can evaluate the key steps of BoNT action: receptor binding, internalization-translocation, and catalytic activity; and therefore could replace the current mouse bioassay. Primary neurons possess appropriate sensitivity to develop potential replacement assays but those potency assays are difficult to perform and validate. This report describes a CBPA utilizing differentiated human neuroblastoma SiMa cells and a sandwich ELISA that measures BoNT/A-dependent intracellular increase of cleaved SNAP25. Assay sensitivity is similar to the mouse bioassay and measures neurotoxin biological activity in bulk drug substance and BOTOX® product (onabotulinumtoxinA). Validation of a version of this CBPA in a Quality Control laboratory has led to FDA, Health Canada, and European Union approval for potency testing of BOTOX®, BOTOX® Cosmetic, and Vistabel®. Moreover, we also developed and optimized a BoNT/A CBPA screening assay that can be used for the discovery of novel BoNT/A inhibitors to treat human disease.

## Introduction

Clostridial neurotoxins bind to nerve terminals and deliver their zinc-endopeptidase (Light Chain, LC) [1] inside the cytosol, where they specifically cleave one of the soluble N-ethylmaleimide-sensitive factor attachment receptor (SNARE) proteins leading to inhibition of neuroexocytosis [2]–[6]. Botulinum neurotoxin serotype A (BoNT/A) causes prolonged, reversible muscle weakness by entering motor nerve terminals and cleaving 9 amino acids from the C-terminus of the SNARE protein SNAP25 (SNAP25_206_) to yield SNAP25_197_ [7], disrupting exocytosis and blocking neurotransmitter release [5], [8], [9]. Because of its potency and specificity for pre-synaptic nerve terminals, BoNT/A is used to treat numerous clinical conditions [10]–[13].

Detection of BoNTs in drug products, contaminated foods, and clinical and environmental samples is challenging because of their potency (i.e., low quantities leading to symptoms). The currently approved method for measuring BoNT biological activity is the mouse LD_50_ (mLD_50_) bioassay [14]–[19], which represents inhibition of the respiratory muscles. The mLD_50_ is highly sensitive (7–20 pg/mL) and has been adopted by all BoNT-based products manufacturers to test drug product potency. The mouse bioassay presents several challenges including assay time required, inability to differentiate between serotypes, sample capacity, and need for highly trained personnel and special animal facilities. Alternatives (i.e., refinements) include the localized muscle paralysis (abdominal ptosis) [20] and Digit Abduction Score assays [21] that are less severe but still require BoNTs injection in animals. Ex vivo alternatives include the rat or mouse phrenic nerve diaphragm [22] and the rat intercostal muscle strips assays [23], [24] that allow several tests from tissues of a single animal. For over 25 years there has been a strong desire to develop in vitro assays that could replace animals or animal tissues [14], [25] and still enable sensitive evaluation of all key steps in BoNT/A action. A sensitive cell-based potency assay (CBPA) is the preferred alternative [16]–[19], [25].

A replacement to the mouse bioassay poses challenging limit of detection (LOD) requirements, in the low pM, because of the minute quantity of BoNT in drug products, and the required sensitivity, accuracy, precision, and reproducibility for Quality Control (QC) standards [14], [18], [25]. Light Chain assays (ELISA [26]–[28], Endopep-MS [29], FRET [30], [31], HPLC-UPLC [32], and DARET [33], [34]) only measure activity of the catalytic domain and cannot detect non-functionality in other BoNT domains (i.e., translocation or binding domains). Previous cell-based assays to screen BoNT inhibitors relied on cells with low toxin sensitivity such as SH-SY5Y [35] or Neuro-2a cells [36], [37]. A reported cell-based FRET assay [30] requires treatments with 50 nM BoNT/A for 48–96 h. Embryonic chicken neurons [38] lack the sensitivity of mammalian neurons. Primary neurons from spinal cord or dorsal root ganglia [39]–[43] are sensitive to BoNT but technically challenging, time-consuming, and highly variable [14], [25]. Sensitive assays that use embryonic stem cell-derived neurons [44]–[47] rely on Western blot read-out with intrinsic variability and their extensive differentiation protocols pose challenges to QC validation. We report here a functional CBPA with differentiated human neuroblastoma SiMa cells [48] that fulfills all the requirements for a replacement assay [14], [25]. It reflects all steps in BoNT/A mechanism of action, its sensitivity (EC_50_∼1-0.4 U/well) is equivalent or better than the mLD_50_, and improving the mLD_50_, it is specific for BoNT/A by measuring SNAP25_197_. It is based on a neuronal cell line and a sandwich ELISA read-out, it is accurate, reproducible, and amenable to QC validation. Moreover, it measures BoNT/A biological activity in BOTOX® (onabotulinumtoxinA) vials.

## Results

### Monoclonal antibody specific for SNAP25_197_

**Figure 1:**
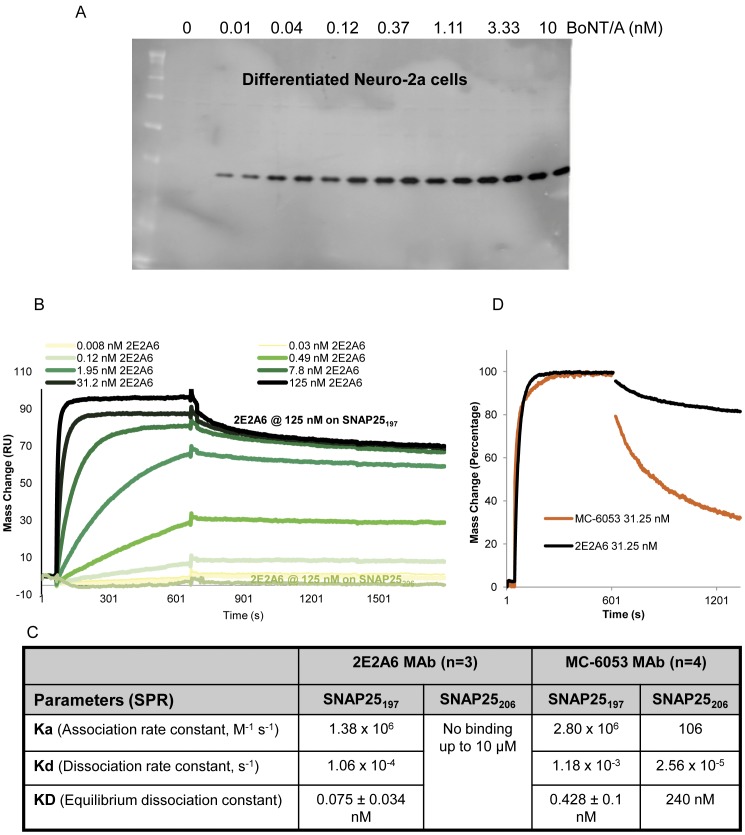
Characterization of anti-SNAP25_197_ monoclonal antibody 2E2A6. Specificity demonstrated by lack of cross-reactivity towards SNAP25_206_. **A.** Neuro-2a cells were treated with BoNT/A (150 kDa) from 0.01 to 10 nM (duplicate wells) for 24 h. Western blot was performed with 2E2A6 antibody (1 µg/mL). No cross-reactivity with SNAP25_206_ (no bands observed in the 0 nM BoNT/A lanes) and no other non-specific bands were detected in the whole blot. **B & C.** Surface plasmon resonance (SPR) was used to characterize the 2E2A6 antibody and to compare the binding affinity and binding kinetics of 2E2A6 and MC-6053. 2E2A6 bound SNAP25_197_ with high affinity and did not bind SNAP25_206_ at any of the concentrations tested up to 10 µM. MC-6053 was able to bind SNAP25_206_ with a K_D_ of 240 nM. **D.** The K_d_ for 2E2A6 is tenfold lower than the K_d_ for MC-6053 as shown in the normalized graph resulting in better affinity.

Enzymatic activity of the BoNT/A-LC generates SNAP25_197_ by cleaving 9 amino acids at the C-terminus of SNAP25_206_ [7]. One of the breakthroughs in the development of the present BoNT/A CBPA was the generation of a monoclonal antibody, 2E2A6 (IgG3.k), recognizing SNAP25_197_. Monoclonal 2E2A6 is highly specific for SNAP25_197_ with no detectable cross-reactivity to SNAP25_206_ in Western blot (Figure 1A), and ELISA (OD_405_ = 0.892 for SNAP25_134–197_ vs. OD_405_ = 0.036 for SNAP25_134–206_ peptides). 2E2A6 has been characterized using Surface Plasmon Resonance (SPR) analysis and compared with a commercial antibody, MC-6053 (Research & Diagnostic Antibodies), claiming to be specific for SNAP25_197_, but that was found to bind SNAP25_206_ with a K_D_ of 240 nM in the SPR analysis demonstrating some cross-reactivity. SPR analysis demonstrated that the 2E2A6 antibody has excellent affinity and specificity for SNAP25_197_ (K_D_ = 0.075 nM for SNAP25_197_ and no binding to SNAP25_206_ at doses up to 10 µM) with a very low dissociation constant (1.06×10^−4^ s^−1^, Figure 1B) that makes it ideal for the development of an assay specific for BoNT/A. This monoclonal antibody ensures a constant and reliable supply of a critical reagent for the CBPA.

### Identification of BoNT/A sensitive SiMa cells

**Figure 2:**
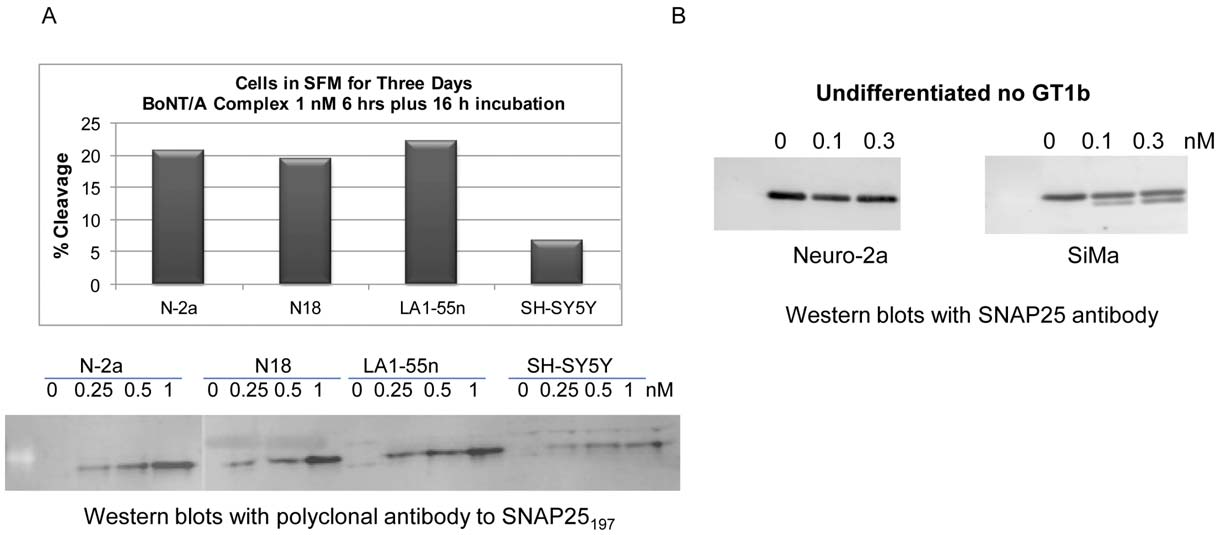
SiMa cells were selected from forty-two cell lines screened for BoNT/A complex uptake. **A.** Example of cell line screening. Differentiated cells were treated with 1 nM BoNT/A for 6 h followed by 16 h incubation to allow for the cleavage of SNAP25. Western blots were performed with an antibody to SNAP25 and the percent SNAP25 cleavage was calculated. Sensitive cell lines Neuro-2a, N18, and LA1-55n produced ∼20% cleavage while SH-SY5Y produced only 7% cleavage. Same cell lines were treated with 0.25, 0.5, and 1 nM BoNT/A. Western blots were performed with anti-SNAP25_197_ polyclonal antibody confirming that SH-SY5Y cells were less sensitive. Cleavage of SNAP25 could be detected with 0.25 nM BoNT/A. **B.** Undifferentiated Neuro-2a and SiMa cells were treated with 0.1 and 0.3 nM BoNT/A complex for 16 h. Western blots were performed with antibody S9684 (Sigma) that recognizes intact and cleaved SNAP25. Under these conditions, SiMa cells produced cleaved SNAP25_197_ at both concentrations while no cleavage was detected in undifferentiated Neuro-2a cells.

**Figure 3:**
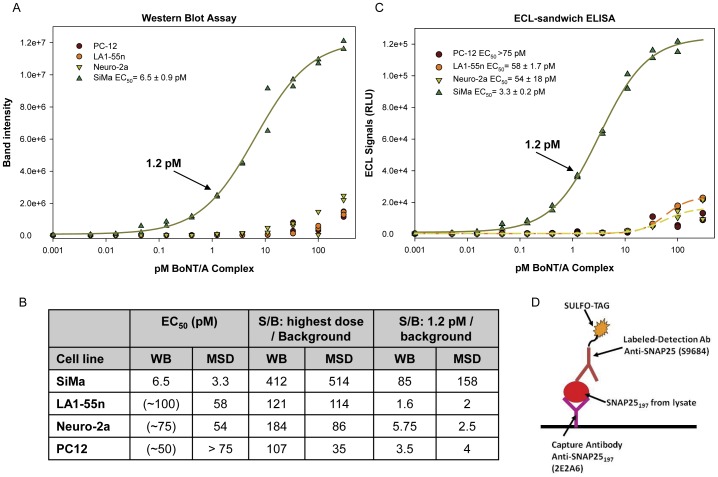
SiMa outperformed other candidate neuronal cell lines in the WB and ELISA assays. Differentiated PC-12, LA1-55n, Neuro-2a, and SiMa cells were treated (0.005–300 pM BoNT/A) for 24 h followed by 48 h incubation in toxin-free medium to allow for SNAP25 cleavage. **A.** Cell lysates were evaluated in a SNAP25_197_-WB and the data fitted to a 4PL model (SigmaPlot v10.0). The EC_50_ = 6.5±0.9 pM (SEM) for SiMa cells while the EC_50_ for the other cell lines could not be calculated. **B.** Summary table containing EC_50_ and signal to background (S/B) at 1.2 and 300 pM for all the candidate cell lines. Historical data utilizing optimized dose-response curves for other cell lines is presented in parenthesis for comparison. **C.** Half of the cell lysates from **A** were tested in the newly developed ECL-sandwich ELISA shown in D. The EC_50_ = 3.3±0.2 pM for SiMa cells while the other two cell lines that reached an upper asymptote produced EC_50_ values of 58 pM (LA1-55n) and 54 pM (Neuro-2a). **D.** Format of the ECL-sandwich ELISA: capture with anti-SNAP25_197_ monoclonal 2E2A6/detection with anti-SNAP25 polyclonal antibody S9684 (Sigma).

Neuronal-derived cell lines were obtained from the American Tissue Culture Collection (ATCC, 24 cell lines), European Collection of Cell Cultures (ECACC, 11 cell lines), and German Collection of Microorganisms and Cell Cultures (DSMZ, 7 cell lines) and screened for their sensitivity to BoNT/A. Differentiated Neuro-2a cells were previously identified as BoNT/A sensitive and served as comparison for screening additional cell lines. The primary screen was performed using BoNT/A complex at 0 and 1 nM with 6 h treatment followed by overnight incubation in toxin free medium to allow for cleavage of SNAP25. Samples were analyzed in Western blots (WB) using anti-SNAP25 antibodies (mAb SMI-81 or pAb S9684) that detect both SNAP25_206_ and SNAP25_197_ bands, allowing the calculation of % cleaved SNAP25. The best cell lines for BoNT/A uptake were Neuro-2a, LA-1-55n, PC12, N18, and SiMa (Figure 2A and Figure 3A). Undifferentiated SiMa cells [48] were more sensitive to BoNT/A than undifferentiated Neuro-2a cells (Figure 2B) with 22.4% SNAP25 cleavage at 0.11 nM and 38% at 0.33 nM BoNT/A after overnight treatment, while no cleavage was detected on undifferentiated Neuro-2a cells under these treatment conditions.

BoNT/A uptake by SiMa cells was compared to the other candidate cell lines using a SNAP25_197_ WB-assay (Figure 3A). Neuro-2a, PC12, LA-1-55n, and SiMa cells were differentiated in collagen IV plates in EMEM serum-free medium with 25 µg/mL GT1b for three days before treatment with BoNT/A complex (0.005 to 300 pM) for 24 h followed by two-day incubation to allow for SNAP25_197_ accumulation. These optimal differentiation and treatment conditions were established previously for Neuro-2a and PC12 cells and were applied to LA-1-55n and SiMa cells. The dose-response curve in figure 3A demonstrates that SiMa cells, with an EC_50_ of 6.5 pM, were very sensitive to BoNT/A (Figure 3B). No EC_50_ values were obtained for the other cell lines since no upper asymptote was achieved. Moreover, a clear signal above background was detected at 0.04 pM (Figure 3A) demonstrating that differentiated SiMa cells possess a high affinity uptake system comparable to primary neurons [39], [42]. SiMa cells displayed better efficacy for BoNT/A uptake at every concentration tested. At the highest BoNT/A dose tested, the signal-to-background (S/B) ratio was 412 for SiMa cells (Figure 3B) with a clear upper asymptote facilitating curve fitting. The S/B indicates the sensitivity of the assay, especially at low doses. At 1.2 pM BoNT/A the S/B ratio was 85 (∼2–6 for the other cell lines). After three days of differentiation in the optimized medium, SiMa cells stopped dividing and acquired morphological characteristics of neurons extending neurites. Additionally, there was an increase in the mRNA levels for SV2A and SV2C, and the differentiated cells expressed SV2B that was not present in undifferentiated cells. A breakthrough in the development of a CBPA for BoNT/A was achieved with the identification of these sensitive SiMa human neuroblastoma cells [48]. Differentiated SiMa cells possess the sensitivity of embryonic spinal cord neurons (eSC) [39]–[41] and embryonic stem cell-derived neurons [44]–[47] and, being an established cell line, they are feasible for use in a QC environment [14].

### Development of an ELISA read-out for the BoNT/A-CBPA

The SNAP25_197_ WB-assay with differentiated SiMa cells described above is the most sensitive assay developed with an established cell line described to date, but Western blot is not viable for high-throughput screening or QC validation. In contrast, sandwich ELISA assays, based on two antibodies which bind to different sites on the antigen, are robust, sensitive, and amenable to validation. The antibody binding affinity for the antigen is usually the main determinant of immunoassay sensitivity; therefore, monoclonal anti-SNAP25_197_ antibodies were used for capture. Twenty-four combinations of capture and detection antibodies were tested for the sandwich ELISA in the MSD® (Meso Scale Discovery) electrochemiluminescence (ECL) detection platform. The combination producing the best results consisted of 2E2A6 monoclonal for capture and anti-SNAP25 polyclonal S9684 (N-terminus) for detection (Figure 3D).

The newly developed ECL-ELISA was compared to the Western blot read-out. Lysates from the four candidate cell lines (half of the lysates from figure 3A) were tested. MSD High Bind plates were coated with 5 µL/well of 2E2A6 at 20 µg/mL. The S9684 polyclonal antibody was labeled with sulfo-tag and 25 µL/well at 5 µg/mL were used for detection. The ECL-ELISA produced excellent signal to background for all the cell lines at the highest dose tested indicating that robust assays could be developed (Figure 3B & 3C). Neuro-2 and LA-1-55n cells produced EC_50_ values of 55 and 58 pM respectively, while the PC12 cells curve did not reach an upper asymptote. Differentiated SiMa cells were very sensitive to BoNT/A with an EC_50_ of 3.3 pM and a plateau at ∼100 pM. The S/B ratios for SiMa cells in the ECL-ELISA were ∼500 at the 100 pM dose and ∼160 at the 1.2 pM dose. All the elements to develop a CBPA for BoNT/A that is sensitive, specific, and amenable to validation were in place.

### Sensitivity and specificity of SiMa cells

**Figure 4:**
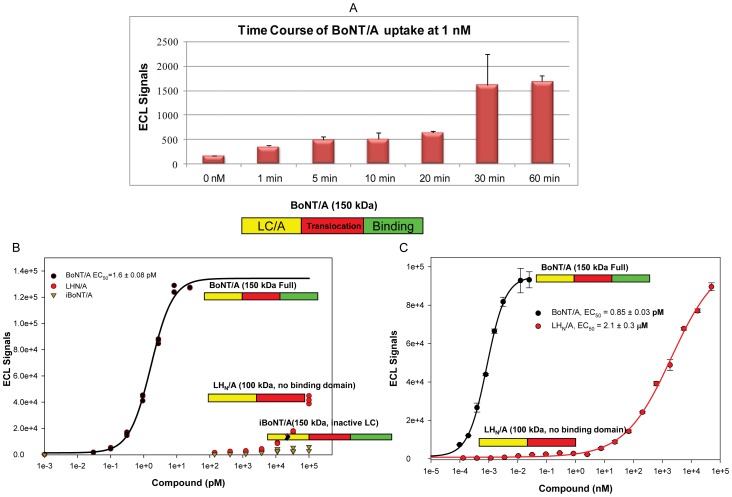
SiMa cells are sensitive and specific for BoNT/A uptake. **A.** Differentiated SiMa cells were treated with 1 nM BoNT/A complex from 1 to 60 min. Cell lysates were evaluated in the ECL-sandwich ELISA. Signal above background was observed at the earliest time points indicating high affinity binding (Error bars = std. dev.). **B.** Specificity of uptake by SiMa cells was demonstrated by comparing the uptake of BoNT/A (150 kDa) to recombinant LH_N_/A (lacking binding domain but comprising the translocation domain and an active light chain) and inactive BoNT/A (iBoNT/A, inactive light chain). No cleavage of SNAP25 was detected with iBoNT/A. Non-specific uptake of LH_N_/A was observed only at doses >10 nM. **C.** Graph comparing specific uptake of BoNT/A complex (at pM concentrations) to a full dose-response of recombinant LH_N_/A (at pM to µM concentrations) with a highest dose of 50 µM. The EC_50_ for the LH_N_/A molecule was 2.1 µM versus 0.85 pM for the fully active BoNT/A.

The hallmarks of high affinity BoNT/A uptake are rapid binding and internalization combined with sensitivity to low toxin concentrations. Those are exemplified in embryonic spinal cord neurons (eSC) exposed to 0.4 pM BoNT/A for two days rendering 50% SNAP25 cleavage [39] and eSC treated with 500 pM BoNT/A for 4 min followed by 2.5 h incubation producing 10–20% SNAP25 cleavage [40]. SiMa cells detect BoNT/A activity at sub-pM toxin concentrations with 24 h treatments (Figure 3). To demonstrate fast uptake, differentiated SiMa cells were treated with 1 nM BoNT/A from 1 to 60 minutes. SiMa cells produced significant cleavage of SNAP25 over background after treatments as short as one minute (Figure 4A).

The specificity of a method defines its ability to measure the analyte of interest and differentiate it from similar compounds. This CBPA is specific to BoNT/A by design since the 2E2A6 monoclonal antibody only recognizes SNAP25_197_. To replace the bioassay, the CBPA must distinguish a fully active neurotoxin from altered or damaged neurotoxins. In the specificity studies, differentiated SiMa cells were treated with recombinant LH_N_/A, lacking the binding domain but containing the Light Chain and Translocation domains, and a recombinant iBoNT/A containing an inactivating mutation in the LC [49] (Figure 4B). SNAP25_197_ was only detected at the higher doses of LH_N_/A tested, suggesting a non-specific internalization of LH_N_/A (signals at 100 nM LH_N_/A were similar to BoNT/A at 0.31 pM) and there was no SNAP25_197_ detected after iBoNT/A treatments. LH_N_/A uptake was at least 60,000 fold lower than 150 kDa BoNT/A (EC_50_ = 1.6 pM). To determine the effects of higher concentrations of LH_N_/A in the SiMa CBA, differentiated SiMa cells were treated with BoNT/A complex (at pM concentrations) or LH_N_/A with a highest dose of 50 µM. The data in figure 4C confirms specificity of the CBPA to fully active toxin and defines the effects of LH_N_/A in the assay at concentrations ∼10^6^ higher than those of active BoNT/A. The EC_50_ for the LH_N_/A molecule was 2.1 µM versus 0.85 pM for the fully active BoNT/A. Moreover, the assay can measure the potency of pure neurotoxin (150 kDa) as well as BoNT/A complex. These results demonstrate that the CBPA mirrors BoNT/A mechanism of action *in vivo*: binding, internalization-translocation, and catalytic activity [14].

### Optimization of the CBPA for BoNT/A

**Figure 5:**
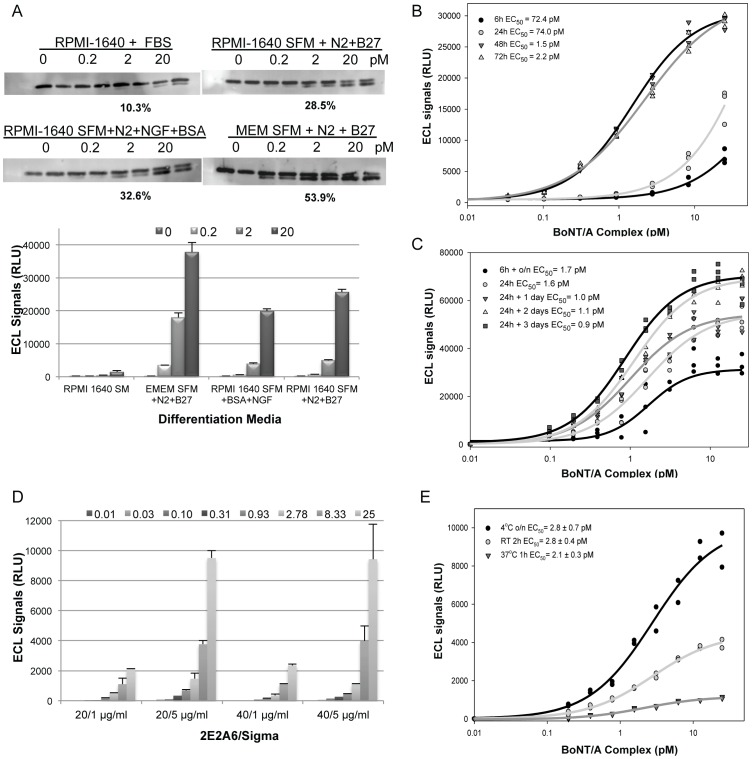
Optimization the SiMa CBPA. **A.** **Optimization of differentiation medium.** SiMa cells were plated in RPMI-1640 with FBS (SM), RPMI-1640 serum-free medium (SFM) supplemented with N2 and B27, RPMI-1640 SFM with N2, BSA and NGF, or EMEM SFM with N2 and B27 for three days. Cells were treated with BoNT/A at 0.2, 2 and 20 pM for 24 h and incubated for 48 h. Lysates were analyzed by WB and the percent cleavage at 2 pM is shown. The same lysates were analyzed in the ECL-ELISA confirming that the EMEM SFM with N2 and B27 supplements was optimal for differentiation (Error bars = std. dev.). Clear signal over background was detected at 0.2 pM. **B.** **Optimization of differentiation time.** SiMa cells were differentiated for 6 to 72 h in EMEM SFM with GT1b, N2 and B27. Cells were treated with BoNT/A from 0.03 to 25 pM for 24 h followed by media change and 48 h incubation. The ECL-ELISA demonstrated that SiMa cells' sensitivity improved with ≥48 h differentiation. **C. Optimization of BoNT/A treatment conditions**. Differentiated SiMa cells were treated with 0.1 to 25 pM BoNT/A for 6 h or 24 h followed by incubation in toxin-free medium for 0, 16, 24, 48, or 72 h. Lysates were analyzed in the ECL-sandwich ELISA. EC_50_ values were similar under all treatment conditions tested but S/B values were different. Optimal BoNT/A treatment to generate a low EC_50_ and high S/B was 24 h followed by 2 or 3 days incubation. **D. Optimization of the ECL-sandwich ELISA conditions.** Concentrations of capture (2E2A6) and detection (S9684) antibodies were optimized. Capture antibody at 20 and 40 µg/mL was tested in combination with detection antibody at 1 and 5 µg/mL to analyze lysates from cells treated with BoNT/A from 0.01 to 25 pM. 2E2A6 at 20 µg/mL spotted in 5 µL combined with S9684 at 5 µg/mL in 25 µL was optimal (Error bars = std. dev.). **E.** Cell lysate incubation time and temperature were evaluated and optimal condition was 16 h incubation at 4°C.

Three major experimental steps require optimization in a CBPA: cell growth and differentiation conditions, drug treatment, and read-out parameters. Factors influencing performance at each step were evaluated individually with BoNT/A uptake as the end-point, measured as the presence of SNAP25_197_, and are summarized in Table 1. The final conditions chosen for the optimized assay were plating 50,000 cells/well in EMEM serum-free medium supplemented with N2 and B27 (Figure 5A) in poly-D-lysine plates for ≥48 h (Figure 5B) followed by 0.004–25 pM BoNT/A treatment for 24 h and two-day incubation in toxin free medium to allow for SNAP25_197_ accumulation (Figure 5C). For the ECL-ELISA, High Bind ELISA plates were spotted with 5 µL of 2E2A6 at 20 µg/mL (Figure 5D), dried and then blocked with 2% ECL (Enhanced Chemiluminescence) with 10% goat serum for 1 h followed by lysate incubation overnight at 4°C (Figure 5E). Sulfo-tag labeled detection antibody was incubated at room temperature for 1 h, reading buffer was added, and the plates were imaged using a SECTOR® Imager 6000.

**Table 1:** Assay Optimization and Standardization.

Parameter	Conditions tested	Optimal
**96-well culture plate**	7 matrices	Poly-D-Lysine
**Differentiation medium**	8 formulations	EMEM SFM plus N2 & B27 supplements and GT1b
**Differentiation time**	6 time points	≥48 h
**Seeding cell density**	4 densities	50,000–100,000 cells/well
**Treatment medium**	5 formulations	Differentiation medium without GT1b
**BoNT/A treatment time**	3 time points	24 h
**Incubation time**	4 incubation times	2 days
**BoNT/A dose range**	0.004–300 pM	0.004 pM (S/B = 4.5)–25 pM
**Amount of capture and detection antibodies**	24 combinations	2E2A6 spotted 5 µL at 20 µg/mL Detection (S9684) 25 µL at 5 µg/mL
**Lysate incubation**	3 temperatures and time	Overnight at 4°C
**Blocking Buffers**	3 buffers	2% ECL blocking with 10% goat serum
**Detection antibody**	2 temperatures plus time	1 h at room temperature
**Edge Effects**	Outside rows and columns	Edge effects. Avoid rows A and H; columns 1 and 12

**Figure 6:**
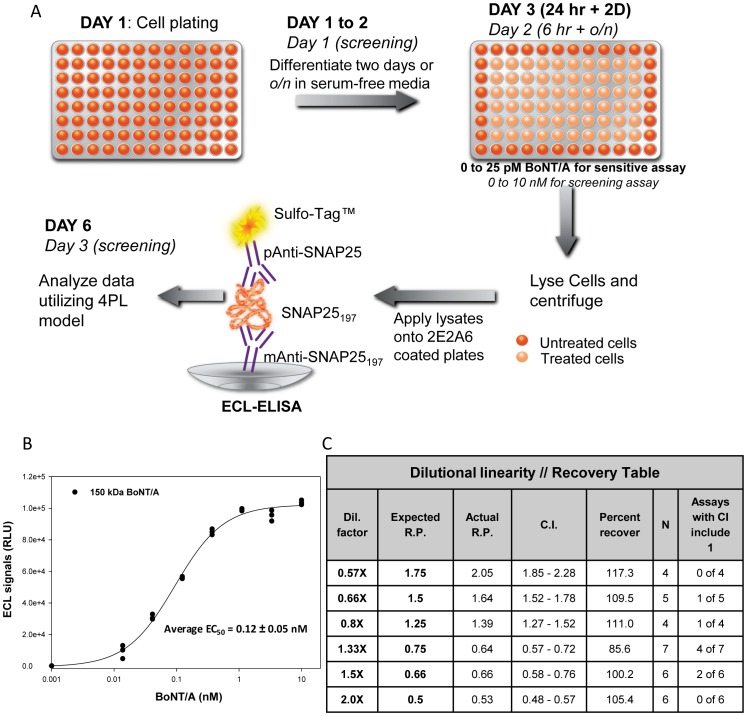
Development of a sensitive screening CBPA. **A.** Protocols for the sensitive and screening (in italic) cell-based potency assays (CBPAs) utilizing differentiated SiMa cells and ECL-sandwich ELISA. **B.** Representative screening CBPA with SiMa cells treated with 0.014–10 nM BoNT/A (150 kDa) for 6 h followed by overnight incubation in toxin-free medium to allow for SNAP25_197_ accumulation. Average EC_50_ of 125 independent assays performed by three operators is shown. **C.** Dilutional linearity-recovery table with concentrations from 1.75 to 0.5 relative potencies (R.P.). The last column exemplifies how many individual assays produced data in which the confidence interval (C.I.) overlapped 1 making that dilution indistinguishable from the reference 1× preparation in that specific assay. Percent recoveries from 86 to 117% demonstrate excellent accuracy of the assay.

Edge effects, a source of variability, are sometimes due to evaporation from wells that are incubated for long periods of time during differentiation or treatment, mainly in the edges of the plates, rows A and H, and columns 1 and 12. Edge effects were evaluated in the tissue culture plates with CellTiter-Glo® to measure cell viability after cell differentiation, BoNT/A treatment, and incubation. The variability (% CV) of rows A and H and columns 1 and 12 were compared to the inner wells in the plate. Edge effects were detected (p<0.001) and the assay was designed avoiding the use of the plate edges (Figure 6A).

System suitability studies (linearity), to ensure that small differences in potency between samples can be detected in the assay, were performed by two operators. To simulate samples with different potencies, samples from 2-fold more concentrated to 0.1 (10-fold) diluted compared to the BoNT/A reference preparation were tested in the assay. For these assays 150 kDa BoNT/A was used. Accuracy was evaluated by calculating the relative potency (RP) and 95% confidence intervals (CI) between the 1× and the concentrated or diluted samples, and by obtaining a percent recovery from the expected and the experimental relative potencies. The data obtained with dilutions from 0.5 (2-fold) to 0.1 (10-fold) (Table S1) demonstrates that the assay can differentiate from 0.5 (2-fold) to 0.16 (6-fold) dilutions, but samples diluted further cannot be distinguished among them. Since this assay is intended to replace the bioassay for product release, the most relevant range is 0.5 to 1.75 relative potency. Results in Table 2 demonstrate that the assay differentiates the 1× sample from both the diluted and the more concentrated samples. Of special interest are the 0.75 and 1.25 relative potencies in which none of the four independent assays performed contained 1× in the 95% CI and were therefore different from the 1× reference preparation. The CBPA can identify samples with 20% difference in potency. Accuracy was excellent with percent recoveries from 96 to 112% in a research laboratory setting. Similar suitability studies were performed with BOTOX® during assay validation in the GMP laboratory leading to assay approval by regulatory agencies.

**Table 2:** System suitability-Sensitive Assay: Excellent accuracy and linearity.

BoNT/A Dilutional linearity//Recovery Table for Sensitive Assay						
Concentration	Expected Relative Potency	Actual Relative Potency	Confidence Interval (CI)	Percent Recovery	Number of assays	Number of assay with CI overlapping 1
**0.57×**	**1.75**	1.69	1.54–1.85	96.3	4	0 of 4
**0.66×**	**1.5**	1.50	1.36–1.66	100	4	0 of 4
***0.8×***	***1.25***	*1.34*	*1.26–1.41*	*106.8*	*4*	*0 of 4*
***1.33×***	***0.75***	*0.85*	*0.78–0.91*	*111.9*	*4*	*0 of 4*
**1.5×**	**0.66**	0.68	0.63–0.73	103.3	4	0 of 4
**2.0×**	**0.5**	0.55	0.52–0.59	111.6	4	0 of 4

To measure the quality and power of the CBPA, the z-prime (Z′ = 1−[3(SD_max_+SD_0_)/(Mean_max_−Mean_0_)) values for 49 assays conducted by three operators were calculated. The Z′ parameter compares the assay dynamic range to data variation and measures how statistically different the experimental values are from the negative control [50]. A Z′ value between 0.5 and 1.0 is defined as an excellent assay suitable for screening with 1.0 being ideal, while a value <0.5 is a marginal assay not suitable for screening. The average Z′ value for the CBPA was 0.82 with values ranging from 0.6 to 0.96 for individual assays, demonstrating an excellent assay.

The CBPA described here is extremely sensitive, accurate, and amenable for screening but requires six days to complete from cell plating to results (3½ days from treatment to data, figure 6A). When screening in process or environmental samples in which botulism patients need to be diagnosed quickly, speed is more important than sensitivity [25]. Therefore, a screening CBPA was designed (Figure 6A) in which cells were plated in optimized differentiation medium overnight and treated the next day with BoNT/A (0.01 to 10 nM) for 6 h followed by an overnight incubation in toxin-free medium to allow for SNAP25_197_ accumulation. To complete the ECL-ELISA in one day, lysates are incubated 2 h at 4°C (cell plating to data two days, treatment to data 1½ days). For both assays hands-on time is approximately 4 h. The screening CBPA is sensitive, EC_50_∼120 pM, and possesses excellent S/B>200 at 120 pM and ∼40 at 10 pM (Figure 6B). The screening assay has been routinely conducted by three operators in our laboratory (125 independent assays performed) with average Z′ of 0.79 (from 0.99 to 0.51) indicating fitness for screening. System suitability and accuracy were evaluated by dilutional linearity studies focusing on relative potencies of 0.5 to 1.75 (Figure 6C). For the 0.5 and 1.75 relative potency samples, all the individual assays differentiated the sample from the 1× reference preparation. Some individual assays for the other dilutions had confidence intervals overlapping 1× and therefore could not be differentiated from the 1× reference preparation running a single assay. However, when at least four independent assays were run and their data combined utilizing PLA software, all the concentrated and diluted samples tested were determined to be different from the 1× reference preparation. Excellent accuracy was obtained with percent recoveries ranging from 85.6 to 117% in a research laboratory setting.

**Figure 7:**
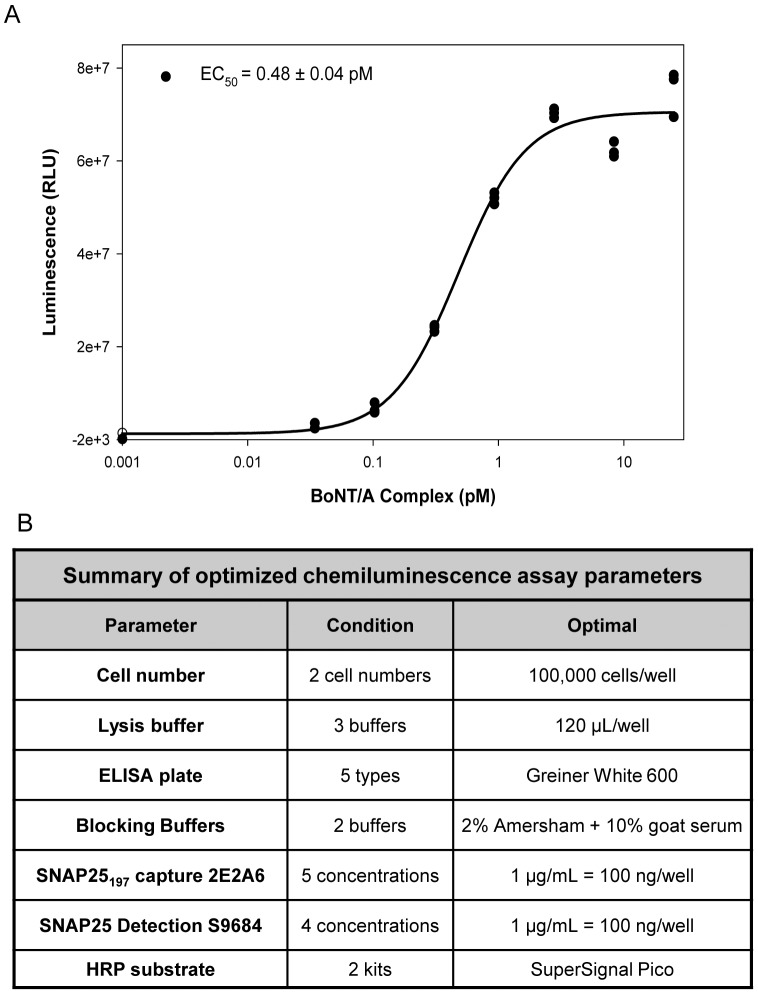
Sandwich ELISA assay with chemiluminescence is as sensitive as the ECL assay. **A.** Example of a representative experiment in which differentiated SiMa cells were treated with BoNT/A at concentrations from 0.03 to 25 pM for 24 h followed by 48 h incubation, and the lysates were evaluated in the optimized chemiluminescence read-out. The results obtained were similar to the ECL read-out (EC_50_∼0.5 to 2 pM), excellent signal to background, and reproducibility of the replicates. **B.** Summary of optimized parameters for the chemiluminescence CBPA. Seven parameters comprising cell culture and ELISA read-out were specifically optimized for this assay by testing several conditions for each parameter.

The CBPA can also utilize standard chemiluminescence as an alternate read-out (Figure 7A) allowing the use of a luminometer to read the plates. The table in figure 7B summarizes the optimized parameters and the final conditions for the BoNT/A sensitive CBPA. A representative run with BoNT/A complex (Figure 7A) demonstrates sensitivity and low variation among replicates. The S/B at 25 pM was ∼400 on average and the S/B at 0.03 pM was ∼10 fold.

### BOTOX® biological activity can be evaluated in the CBPA

**Figure 8:**
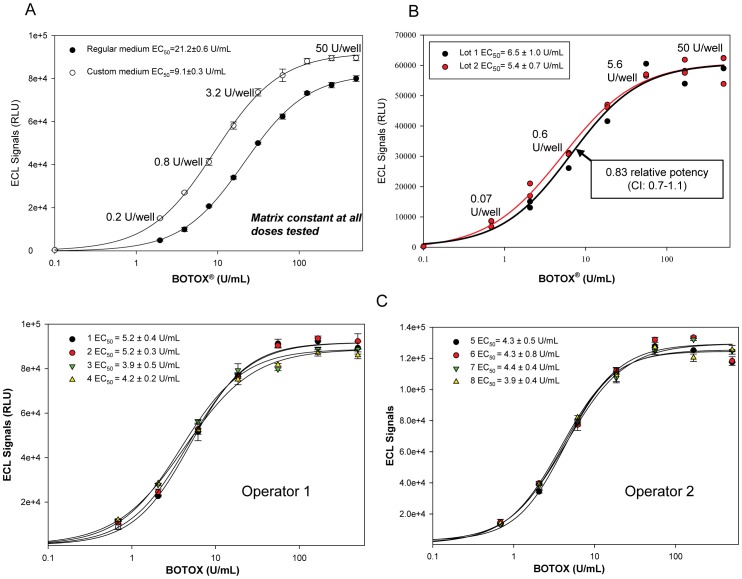
The CBPA can measure BoNT/A biological activity in BOTOX® vials. **A.** A custom medium to reconstitute BOTOX® vials (the nominal value of 100 U was used) was designed to overcome the hypertonicity caused by NaCl present in the formulation. The matrix for the subsequent dilutions was kept constant. Performance of the assay was improved resulting in better sensitivity (EC_50_ = 0.9 U/well) and higher efficacy of uptake. **B.** Two different lots of BOTOX® (the nominal value of 100 U was used) were evaluated in the CBPA. Data was analyzed in PLA 2.0 resulting in 0.82 relative potency (CI: 0.7–1.1) indicating similar potency of both lots. **C.** A single lot of BOTOX® was tested by two operators (n = 8 and n = 9 independent experiments respectively), four runs for each operator are shown. Relative potencies were similar for all the tests performed demonstrating consistent performance by two operators.

BOTOX® (onabotulinumtoxinA) contains 0.9 mg NaCl and 0.5 mg human serum albumin (HSA) in each 100 U vial (Units in each vial are determined utilizing Allergan's validated mLD_50_ assay, are specific to BOTOX®, and are not interchangeable with other commercial Botulinum neurotoxin preparations). Reconstitution of BOTOX® (the nominal value of 100 U was used) with isotonic culture medium (BoNT/A concentration 500 U/mL or ∼25 pM) results in a hypertonic medium with detrimental effects on cells in culture. Testing each excipient separately in the assay demonstrated that only NaCl at the 500 U/mL dose negatively affected SiMa cells and reduced toxin uptake and/or cell viability. To circumvent hypertonicity, a custom EMEM medium was designed and used to reconstitute 100 U BOTOX® vials (500 U/mL or ∼25 pM). The matrix was kept constant for all concentrations along the dose-response curve by adding NaCl and HSA to the dilution medium to match the amount of excipients present at 500 U/mL. The assay became more sensitive with an EC_50_ of 9 U/mL (0.9 U/well) and an S/B ratio of 50 at 2 U/mL (0.2 U/well) (Figure 8A). The data clearly demonstrates that the CBPA can measure BoNT/A biological activity in a formulated product with identical sensitivity to the mouse bioassay. In order to determine the utility of the assay with pharmaceutical preparations, two lots of BOTOX® were tested in the research laboratory (Figure 8B) generating a relative potency of 0.83 with a confidence interval of 0.7 to 1.1 indicating that the potency of the two lots is indistinguishable since the confidence interval included the number one. Moreover, to determine the reproducibility of the assay by several operators, a single lot of BOTOX® was tested in the optimized assay in several plates by two operators in the research laboratory (Figure 8C). The average EC_50_ was 3.96±0.16 U/mL for operator 1 (n = 8) and 4.44±0.16 U/mL (n = 9) for operator 2. To compare the performance of the CBPA when different forms of BoNT/A are tested, the ECL-ELISA assay with differentiated SiMa cells was used to test the biological activity of BoNT/A complex, 150 kDa neurotoxin, and BOTOX® in different plates (Figure S1). The EC_50_ values obtained in the assays were 1.1±0.3 pM for BoNT/A complex, 1.5±0.2 pM for 150 kDa BoNT/A, and 1.35±0.05 pM for BOTOX®, demonstrating that when BOTOX® was diluted in the custom medium, designed to overcome the effects of the excipients present in the formulation, BoNT/A biological activity in the formulated product can be accurately measured. In conclusion, a cell-based potency assay with excellent sensitivity, specificity, accuracy, and precision has been developed to fully replace the mouse bioassay to measure BoNT/A biological activity.

## Discussion

The sensitive BoNT/A CBPA reported here utilizing differentiated human neuroblastoma SiMa cells and an ECL sandwich ELISA satisfies all the requirements for a fully in vitro replacement of the mouse bioassay for BoNT/A [14], [16]–[19], [25] and fulfills our long-standing commitment to the “3R” principles of refinement, reduction and eventual replacement of the bioassay [18] in the final product release. The replacement CBPA for BoNT/A potency testing has a sensitivity similar to the mouse bioassay (EC_50_∼1-0.4 U/well) and is specific for BoNT/A, by the use of monoclonal antibodies specific for SNAP25_197_. The assay utilizes a stable cell line of neuronal origin [48] that can be differentiated in 48 h and a sensitive sandwich ELISA read-out that can be validated in a QC laboratory. This CBPA represents the multi-step pharmacological mode of action of BoNT/A at pre-synaptic terminals [3], [4], [8]; it is accurate, robust, reproducible, amenable to validation, and can measure BoNT/A biological activity in pharmaceutical preparations (containing less than a nanogram of BoNT/A formulated with excipients).

For over 25 years there has been a strong desire to replace the mouse bioassay with a fully in vitro assay that enables sensitive evaluation of all key steps in BoNT/A action [14], [18], [25]. It was the dogma that continuous cell lines lacked the sensitivity necessary to develop an assay that could replace the mouse bioassay [47], but at the same time the use of primary neurons or embryonic cell derived neurons pose their own challenges as they have to be freshly derived from animal tissue [39]–[42] or they require complicated protocols and long time to be fully differentiated [44]–[46]. Moreover, when replacing a bioassay approved by regulatory agencies with a new in vitro assay, the sensitivity of the method is not the only consideration as the assay has to be validated and cross-validated against the mouse bioassay [18], [25]. Our team set in place a rigorous evaluation process of continuous cell lines for their sensitivity to BoNT/A that culminated in the identification of several sensitive cell lines that could be amenable for developing potency assays, with SiMa cells being the most sensitive even when undifferentiated (Figure 2). However, to achieve the sensitivity needed to replace the mouse bioassay (pM concentrations), optimization of the cells' growth and differentiation conditions was essential. After the optimization process with Neuro-2a, PC12, LA1-55n, and SiMa cell lines, we achieved great sensitivity with EC_50_ values in the mid and low pM (Figure 3) that were excellent to develop BoNT/A activity assays. A breakthrough was achieved with the identification of the SiMa cells that were very sensitive (EC_50_ = 6.5 pM in WB and 3 pM in ELISA) to BoNT/A and produced excellent S/B at all doses tested, especially at sub-pM concentrations, comparable to the primary and embryonic cell derived neurons [40], [41], [44]–[47] while providing a continuous and reliable source of cells (allowing preparation of cell banks) for the CBPA.

The development of a read-out for the cell-based assay that could be validated in a QC environment was essential since Western blots, with intrinsic variability, are difficult to validate. Sandwich ELISA assays are robust, sensitive, and amenable to validation. The antibody binding affinity for the antigen is usually the main determinant of immunoassay sensitivity. The second breakthrough was achieved with the generation of a highly specific high affinity anti-SNAP25_197_ monoclonal antibody. The 2E2A6 antibody, displaying high affinity and a very low dissociation constant (Figure 1) was ideal to capture cleaved SNAP25_197_ from cell lysates treated with BoNT/A. The high specificity for SNAP25_197_ resulted in extremely low background signal from untreated lysates; excellent signal to background ratios, even at femtomolar amounts of BoNT/A; and Z′ values that support the use of the assay for screening.

A very sensitive CBPA is needed for measuring BoNT/A biological activity in pharmaceutical products and in other situations in which the toxin is present in very low concentrations. But during outbreaks when patients are waiting for a diagnosis or for research to identify BoNT/A inhibitors, the speed of the assay becomes more important. Our team was able to develop a shorter, but yet sensitive assay (Figure 6, EC_50_ = 120 pM) that can produce reliable measurement of BoNT/A activity in only 2½ days. Additionally, we rigorously optimized and characterized both CBPAs (sensitive and screening, figure 6A). For the sensitive CBPA we optimized and standardized twenty parameters comprising cell growth and differentiation, BoNT/A treatment, and ECL-ELISA (Table 1). Moreover, dilutional linearity/recovery experiments performed with both assays by three operators demonstrated excellent accuracy and precision (Tables 2 and S1). The optimized CBPA was slightly modified to test BOTOX® by designing a custom medium to overcome the adverse effects of the excipients found in the formulation (Figure 8A). In conclusion, this is the first CBPA utilizing an established cell line reported to measure BoNT/A biological activity in BOTOX® possessing sensitivity equal or superior (EC_50_∼1-0.4 U/well) to the mouse bioassay and being specific for BoNT/A by design. Further development, validation, and cross-validation of a version of this CBPA has resulted in FDA, Health Canada, and European Union approval for use in the potency testing of BOTOX® (onabotulinumtoxinA), BOTOX® Cosmetic, and Vistabel®.

## Materials and Methods

### Cell Lines and Growth Conditions

#### Unless otherwise stated, tissue culture reagents were from Invitrogen, Carlsbad, CA

PC-12- Rat pheochromocytoma (CRL-1721; ATCC) were cultured in collagen IV plates in ATCC's recommended medium. *Differentiation medium:* RPMI medium with 2 mM GlutaMAX™, 1× B27 supplement, 1× N2 supplement, 10 mM HEPES, 1 mM Sodium Pyruvate, 50 ng/mL NGF, 100 U/mL Penicillin, and 100 µg/mL Streptomycin. Neuro-2a- Murine neuroblastoma (CCL-131; ATCC) were cultured in Costar flasks in ATCC's recommended medium. *Differentiation medium:* EMEM with 2 mM GlutaMAX™, 0.1 mM NEAA, 10 mM HEPES, 1× N2 supplement, and 1× B27 supplement. SH-SY5Y- Human neuroblastoma (94030304; ECACC) were cultured in Costar tissue culture flasks in ECACC's recommended medium. *Differentiation medium:* EMEM with 2 mM GlutaMAX™, 0.1 mM NEAA, 10 mM HEPES, 1× N2 supplement, and 1× B27 supplement. N18- Mouse neuroblastoma×Rat glioma hybrid (88112301; ECACC) were cultured in Costar tissue culture flasks, 162 cm^2^ (CLS3150; Corning). *Growth medium:* DMEM with 2 mM glucose, 2 mM glutamine, and 10% heat-inactivated FBS. LA1-55n- human neuroblastoma (06041203; ECACC) were cultured in Costar flasks in ECACC's recommended medium. SiMa- human neuroblastoma (ACC 164, DSMZ) were cultured in collagen IV flasks in DSMZ's recommended medium. *Differentiation medium:* Minimum Essential Medium with 2 mM GlutaMAX™ I with Earle's salts, 0.1 mM Non-Essential Amino-Acids, 10 mM HEPES, 1× N2 supplement, and 1× B27 supplement. For optimal differentiation, PC-12, Neuro-2a, LA1-55n, and SiMa cells were plated in 96-well plates at 5×10^4^ cells/well in 100 µL differentiation medium for three days.

### Monoclonal antibody to SNAP25_197_

Murine monoclonal antibodies specific for SNAP25_197_ were generated with the (C)DSNKTRIDEANQ peptide utilizing standard immunization protocols. Antibodies to SNAP25_197_ were screened in WB and ELISA. Antibodies were affinity purified from ascites before use in the SPR and ELISA assays.

### Surface Plasmon Resonance Binding Analysis

Experiments were performed on a BIAcore 3000 instrument (GE Healthcare). Ligands, SNAP25_134–197_ and SNAP25_134–206_ peptides, were immobilized on a CM5 chip using amine coupling. Analytes, Anti-SNAP25_197_ 2E2A6 (1.1 mg/mL stock solution, AGN) or MC-6053 (15 µg/mL stock solution, Protein A purified, Research and Diagnostics Antibodies) antibodies, were injected at 0–125 nM. Sensorgram curves were fitted to a 1∶1 Langmuir binding model (BIAevaluation 3.0 software, GE Healthcare). The association, k_a_ (1/Ms), the dissociation, k_d_ (1/s) and the equilibrium, K_D_ (K_D_ = k_d_/k_a_) (M) constants were determined.

### BoNT/A treatment

Cells were treated with BoNT/A complex, 150 kDa BoNT/A (Metabiologics, Madison, WI), recombinant LH_N_/A or iBoNT/A, or with BOTOX® (Allergan, Irvien, CA) for different amounts of time as specified in each assay. The medium containing BoNT/A was then replaced with fresh medium without toxin and cells were incubated for additional periods of time as specified in each assay. Each concentration of BoNT/A was tested in triplicate.

### Cell Lysis and Western Blot Analysis

Cells were washed once with PBS and lysed in freshly prepared Triton X-100 Lysis Buffer (50 mM HEPES, 150 mM NaCl, 1.5 mM MgCl_2_, 1 mM EGTA, 1% Triton X-100, and one tablet of EDTA-free protease inhibitors) on ice for 20 min. Lysed cells were centrifuged in the plate at 4000 rpm for 20 min at 4°C. Western blots (WB) for detection of SNAP25_206_ and SNAP25_197_ or only SNAP25_197_ were described previously [51]. For WB analysis the lysates were transferred to a 96-well PCR plate, 2× SDS-PAGE loading buffer (Invitrogen) was added, and the plate was heated to 95°C for 5 minutes. The gels were run in 1× MOPS-SDS running buffer (Invitrogen) at 200 V for 55 min. Proteins were transferred to nitrocellulose membranes (Bio-Rad) pre-wet in Western blot transfer buffer (Invitrogen) containing 20% Methanol (Burdick and Jackson). The Western blot transferred at 800 mA for 2 h using the TE-62 transfer cell apparatus (GE Healthcare). Blots were blocked in 2% ECL blocking agent (GE Healthcare) in 1× TBS/0.1% Tween 20 (Bio-Rad) (TBS-T) for 1 h at room temperature. The following primary antibodies were used: anti-SNAP25_197_ polyclonal antibody [51] diluted 1∶1000, SMI-81 antibody (Sternberger Monoclonals Inc) diluted 1∶5000 to evaluate the intact and cleaved SNAP25 products (i.e. SNAP25_206_ and SNAP25_197_ were detected). Antibodies were diluted in 2% blocking agent/TBS-T buffer and incubated overnight at 4°C with gentle shaking. Secondary antibodies were anti-rabbit or anti-mouse IgG H+L HRP conjugated (Invitrogen) diluted 1∶5,000 or 1∶10,000 in 2% blocking agent/TBS-T buffer for 1 h at room temperature. The membranes were washed and exposed for 5 min to ECL Plus Western Blotting System Detection Reagents (GE Healthcare). Chemifluorescence was captured by scanning the blots in the Typhoon 9410 Imager (GE Healthcare) at λex 452/λem 520. The intensity of the gel bands were calculated using ImageQuant TL software (GE Healthcare). The data was analyzed using SigmaPlot v 10.0 (Systat Software Inc.) or PLA2.0 (Stegmann Systems). Intensity values were plotted against concentration of BoNT/A in log scale and fitted to a 4-parameter logistics function (Y = Y_0_+a/[1+(X/X_0_)^b^]) without constraints. Based on the fitted curves the EC_50_ values, corresponding to “X_0_”, were determined.

### ECL and chemiluminescence sandwich ELISA

For the ECL sandwich ELISA, MSD High Bind plates (Meso Scale Discovery) pre-spotted with anti-SNAP25_197_ MAb 2E2A6 were blocked with 150 µL blocking buffer for 1 h at RT. After blocking, the buffer was discarded and 25 µL of cell lysate were added to each well of the plate followed by incubation as detailed in text. Plates were washed with PBS-T, and SULFO-TAG NHS-Ester labeled detection pAb anti-SNAP25 antibody (Antibody to N-terminus of SNAP25, Cat# S9684, Sigma) in diluent buffer was added. Plates were sealed and shaken at room temperature for 1 h, washed with PBS-T, and 150 µL of 1× Read Buffer was added per well. Plates were immediately read on the SI6000 Image plate reader. For the chemiluminescence sandwich ELISA, white plates (Greiner) were coated with 100 µL/well of anti-SNAP25_197_ 2E2A6 MAb at 4°C overnight. Plates were blocked with 2% ECL blocking with 10% goat serum for 1 h at RT. Fifty microliters of cell lysate were added to each well and the plates were incubated at 4°C. S9684 anti-SNAP25 pAb conjugated with HRP was used for detection. The plates were developed with SuperSignal ELISA Pico 1∶1 mixture (Pierce) and read at 395 nm on a Luminometer (Molecular Devices). Data was fitted to a 4PL model as detailed above.

## Supporting Information

Figure S1 **The ECL-ELISA CBPA with SiMa cells can be used to test the biological activity of BoNT/A complex, 150 kDa neurotoxin, and BOTOX®.** Comparison of CBPAs performed with **A.** BoNT/A complex, **B.** 150 kDa BoNT/A, and **C.** BOTOX® (the nominal value of 100 U was used) utilizing the optimized assay conditions. The EC_50_ values obtained in the assays are very similar demonstrating that the assay is robust, versatile, and can detect BoNT/A biological activity at very low concentrations in the presence of formulation excipients.
(TIF)

Table S1 (TIF)
