# Supplementary figures and images for: Botulinum Neurotoxin Serotype a Specific Cell-Based Potency Assay to Replace the Mouse Bioassay
Source: PLoS One. 2012 Nov 21;7(11):e49516. doi: 10.1371/journal.pone.0049516 (PMC3504020; doi:10.1371/journal.pone.0049516)

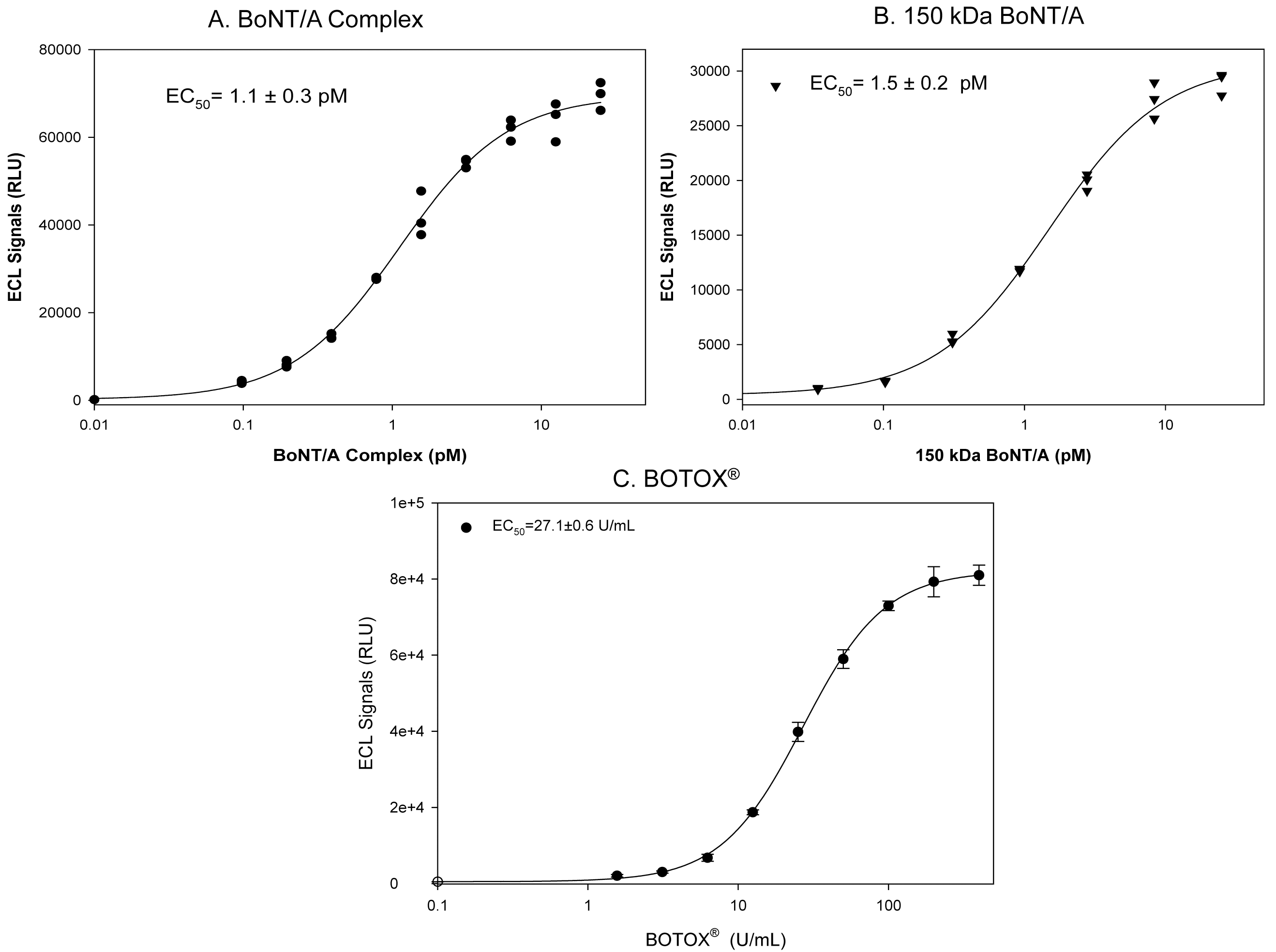

Supplement: Figure S1 — The ECL-ELISA CBPA with SiMa cells can be used to test the biological activity of BoNT/A complex, 150 kDa neurotoxin, and BOTOX®. Comparison of CBPAs performed with A. BoNT/A complex, B. 150 kDa BoNT/A, and C. BOTOX® (the nominal value of 100 U was used) utilizing the optimized assay conditions. The EC50 values obtained in the assays are very similar demonstrating that the assay is robust, versatile, and can detect BoNT/A biological activity at very low concentrations in the presence of formulation excipients. (TIF) [file pone.0049516.s001.tif]

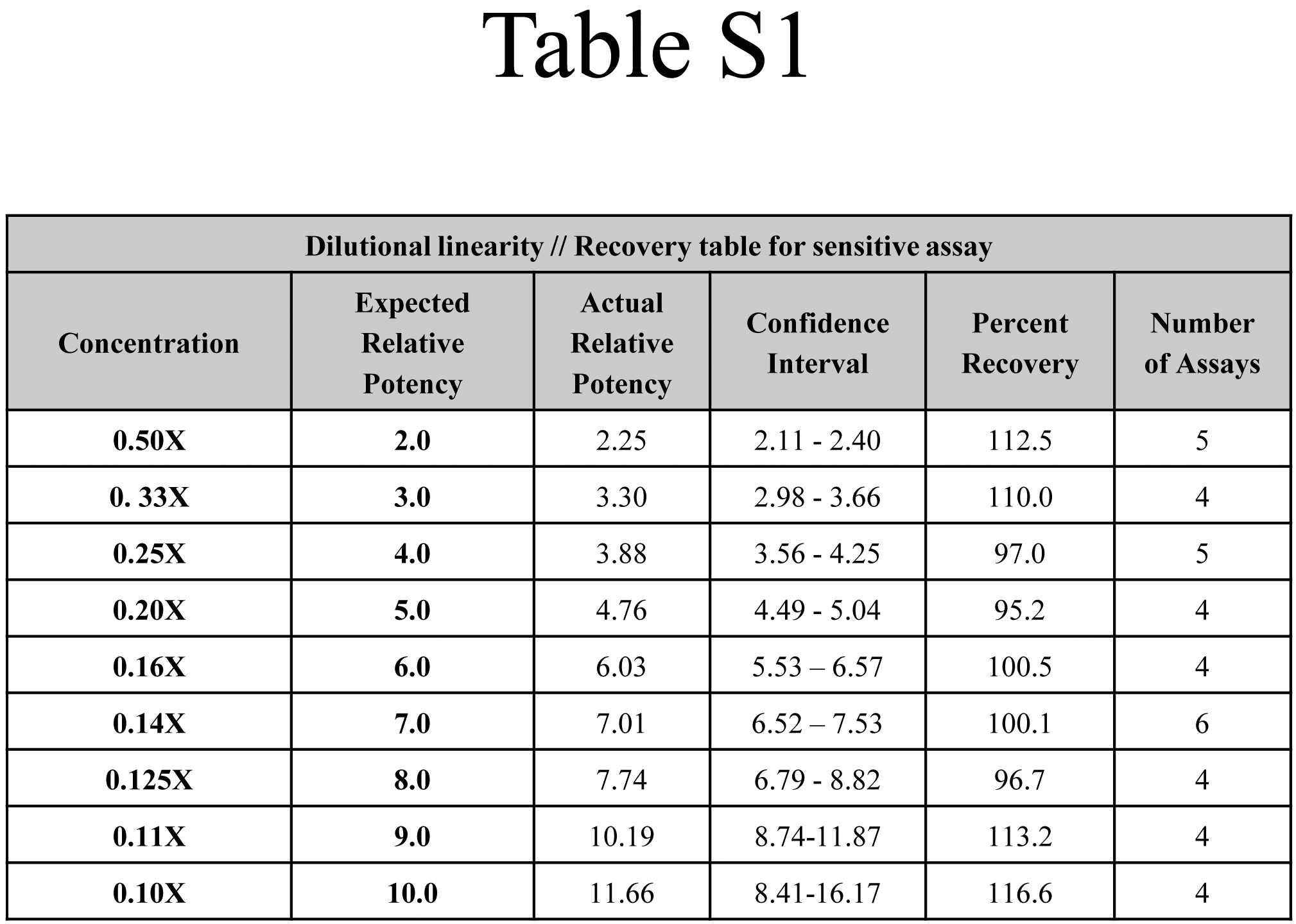

Supplement: Table S1 — (TIF) [file pone.0049516.s002.tif]
